# Cloning and Transcriptional Activity Analysis of the Bovine *CDH11* Gene Promoter: Transcription Factors Sp1 and GR Regulate Bovine *CDH11* Expression

**DOI:** 10.3390/ani15091217

**Published:** 2025-04-25

**Authors:** Zhanxin Liu, Yanbin Bai, Zongchang Chen, Yanmei Niu, Xue Jia, Liang Li, Xiaolan Zhang, Binggang Shi, Zhidong Zhao, Jiang Hu, Yuzhu Luo, Jiqing Wang, Xiu Liu, Shaobin Li, Fangfang Zhao

**Affiliations:** Gansu Provincial Key Laboratory of Herbivore Biotechnology, College of Animal Science and Technology, Gansu Agricultural University, Lanzhou 730070, China; liuzx@st.gsau.edu.cn (Z.L.); baiyb@st.gsau.edu.cn (Y.B.); chenzongc@st.gsua.edu.cn (Z.C.); niuym@st.gsau.edu.cn (Y.N.); jiax@st.gsau.edu.cn (X.J.); liliang@st.gsau.edu.cn (L.L.); zhangxl@gsau.edu.cn (X.Z.); shibg@gsau.edu.cn (B.S.); huj@gsau.edu.cn (J.H.); luoyz@gsau.edu.cn (Y.L.); wangjq@gsau.edu.cn (J.W.); liux@gsau.edu.cn (X.L.); lisb@gsau.edu.cn (S.L.); zhaofangfang@gsau.edu.cn (F.Z.)

**Keywords:** bovine, *CDH11* gene, transcriptional regulation, skeletal muscle

## Abstract

Growth rate of bovine skeletal muscle has a major impact on beef yield. Cadherin-11 (*CDH11*) was found to be a potential candidate gene for growth and development in beef cattle. This study confirmed the high expression of *CDH11* in bovine *longissimus dorsi* using a tissue expression analysis. To understand the transcriptional regulation mechanism of *CDH11* gene, we constructed a double luciferase vector for the promoter region of *CDH11* gene and determined that the core transcriptional regulatory region was located at −129/+55 bp relative to the transcription start site (TSS). In addition, we confirmed that skeletal muscle growth and development-related transcription factor-specific protein 1 (SP1) and glucocorticoid receptor (GR) bind to the *CDH11* gene promoter region at −36/−27 bp and −20/−11 bp, respectively, to regulate *CDH11* expression. These interactions provide valuable information for understanding the mechanism of transcriptional regulation of the bovine *CDH11* gene during muscle growth and development.

## Simple Summary

Skeletal muscle is an important meat-producing tissue of beef cattle, accounting for about 40% of the carcass weight of livestock and poultry, and its growth and development directly affect the economic benefits of animal husbandry. Maximizing the role of functional genes through molecular means is important for breeding beef cattle with faster growth and better body shape. A genome-wide association study (GWAS) revealed that the *CDH11* gene plays an important role in the growth and development of beef cattle, but the specific transcriptional regulation mechanism of the promoter region of this gene is still unclear. The present study constructed a segment-by-segment deletion vector for the promoter region of the *CDH11* gene and then used C2C12 cells to detect dual luciferase activity in order to screen out the core transcriptional regulatory region. The key transcription factors in this region were then predicted using biological information. Finally, these predictions were confirmed using targeted mutagenesis, an electrophoretic mobility shift assay, and an RNA interference assay. The results of these assays showed that the key GR and SP1 transcription factors have an important influence on the transcriptional activity of the *CDH11* gene.

## 1. Introduction

Muscle development plays a pivotal role in determining the growth rate of beef cattle. Skeletal muscle, which constitutes the major component of muscular tissue in animals, exerts a direct influence on the economic aspects of intensive beef cattle production [1,2]. The process of skeletal myogenesis is intricate and involves coordinated actions among multiple cell types. It is noteworthy that this process is finely regulated by different signaling pathways and transcription factors at various stages, encompassing prenatal myoblast proliferation, fusion for differentiation into myofibers, and postnatal myofiber hypertrophy [3,4,5,6]. Although genome-wide association studies (GWASs) have identified numerous genes associated with growth traits in beef cattle, limited investigations have been conducted regarding the epigenetic mechanisms underlying these specific genes and their interactions with specific binding transcription factors [7].

Cadherin-11 (*CDH11*) is a transmembrane protein gene that encodes type II classical calreticulin, playing a crucial role in organismal growth and development primarily through the trans dimerization of extracellular calreticulin structural domain 1 to generate calreticulin complexes [8,9]. Based on the results of early GWASs and RNA-seq studies, the *CDH11* gene may have an important role in regulating economic traits, such as height, body size, and calf weaning weight, in Charolais beef cattle and showed significant differential expression in Yunling and Leijong cattle growth. These findings suggest that the *CDH11* gene may have a key function in the regulation of bovine muscle tissue growth and development [10,11,12]. The proliferation and maturation of myoblasts are pivotal processes in myogenesis. Previous studies have reported the involvement of the *CDH11* gene in the growth and development of myoblasts. In chicken cells, miR-205a regulates the *CDH11* gene involved in the TGFβII pathway. This complex regulates the TGF-β1 pathway at both transcriptional and post-transcriptional levels, thereby enhancing the proliferation and differentiation of QM7 cells along with primary myoblast [13]. Moreover, *CDH11* plays a crucial role in myogenic differentiation by acting as a mesenchymal calcineurin mediating adhesion between homologous cells. When high-density myoblasts come into contact with each other, intercellular adhesion occurs via calcineurins, which promote tight junction formation, leading to fusion of mononuclear myoblasts into multinucleated myotubes. This process induces myoblast differentiation, resulting in muscle fibers formation and ultimately contributing to increased meat production [14,15,16]. Additionally, apart from nutritional and environmental factors influencing animal growth and development, genetic regulation involving transcription factors also plays an important role specifically for skeletal muscle growth and development [17,18,19]. However, research on the regulatory mechanisms associated with *CDH11* has primarily focused on human diseases. A case in point is that FOXF1 regulates TGF-β1-induced damage in BEAS-2B cells by modulating *CDH11* gene-mediated Wnt/β connexin signaling [20]. Furthermore, it has been discovered that the transcription factor homology box C8 (HOXC8) specifically binds to the promoter region of the *CDH11* gene and maintains high levels of *CDH11* gene expression in breast cancer [21].

Given the critical role of the *CDH11* gene in the growth and development of beef cattle, coupled with limited knowledge regarding its involvement in the transcriptional regulation of skeletal muscle growth and development, the aim of this study was to identify the core promoter region of the bovine *CDH11* gene and to elucidate the roles of transcription factors associated with musculogenesis in controlling the transcription of the *CDH11* gene. These findings provide a theoretical basis for future studies on the functional significance of the *CDH11* gene in muscle growth and development and genetic improvement of beef cattle.

## 2. Materials and Methods

### 2.1. Sample Collection

Samples utilized for the experiment were heart, liver, spleen, lung, kidney, *longissimus dorsi* muscle, and subcutaneous fat samples from three healthy 1-year-old bulls, which were obtained from the livestock farm of Gansu Agricultural University (Lanzhou, China). Subsequently, these samples were rapidly immersed in liquid nitrogen and subsequently stored at −80 °C.

### 2.2. Isolation of DNA and the Detection of CDH11 Gene mRNA Expression

This genomic DNA from the *longissimus dorsi* muscle was isolated using the TRlzol method described by Adam Kotorashvili et al. [22]. Total RNA was extracted from tissues and cells using AG RNAex Pro Reagent (Accurate, Biotechnology, Hunan, China). The integrity of the RNA samples was assessed through agarose gel electrophoresis (JunYi, Beijing, China), while concentration and purity were determined using a Nano Dro8000 spectrophotometer (ND8000-GL, NanoDrop Technologies, Wilmington, NC, USA). Subsequently, cDNA synthesis was performed according to the TransScript One-Step gDNA Removal and cDNA Synthesis Super Mix (AT311, Transgen, Beijing, China).

Fluorescent quantitative primers were designed based on the bovine *CDH11* gene family published by UCSC Genome Browser (https://genome.ucsc.edu, accession number: NM_001081624.2, Accessed: April 2024) using Clone Manage software (Table S1). Quantification of mRNA expression levels was carried out with the Perfect Start Green qPCR Super Mix kit (AQ601, Transgen, Beijing, China) The reaction mixture consisted of 20 μL total volume containing 0.4 μL each of forward and reverse primers, 10 μL of 2× PerfectStart Green qPCR Super Mix, 0.4 μL of Passive Reference Dye (50×), and 2 μL Template and 6.8 μL of Nuclease-free Water. qRT-PCR reaction conditions included pre-denaturation at 94 °C for 30 s; denaturation at 94 °C for 5 s; and annealing at 60 °C for 30 s, with a total of 42 cycles. Gene expression levels were normalized to Glyceraldehyde-3 phosphate dehydrogenase (*GAPDH*) expression and calculated using the −2^ΔΔCt^ method.

### 2.3. Proximal Initiation Sub-Bioinformatics Analysis and Prediction

The 5′UTR proximal −1855/+ 55 bp DNA sequence of the bovine *CDH11* gene (GenBank: NM_001081624.2) was obtained from the UCSC Genome Brower Home database (https:/genome.ucsc.edu). A neighbor-joining method through Mega 11 (Philadelphia, USA) was employed to constructed a phylogenetic tree. Uniport (https://www.uniprot.org, Accessed: April 2024) was utilized to analyze protein homology of the *CDH11* gene across *Bos mutus*, *Capra hircus*, *Ovis aries*, *Sus scrofa*, *Homo sapiens*, *Mus musculus*, and *Gallus gallus*. CpG sites were predicted using Methprimer (http://www.urogene.org/cgi-bin/methprimer/methprimer.cgi, Accessed: April 2024), while the transcription start sites were predicted using BDGP (https://www.fruitfly.org, Accessed: April 2024). Furthermore, Gene Regulation (http://gene-regulation.com, Accessed: April 2024) combined with Jaspar (https://jaspar.elixir.no, Accessed: April 2024) predictions were used predict transcription-factor-binding sites within the core transcriptional regulatory region of the promoter.

### 2.4. Construction of Luciferase Deletion Vector Plasmid in the 5′ UTR Proximal Promoter Region of Bovine CDH11 Gene

In this experiment, the purified fragments were digested with NheI and XhoI (JN301, JX201, Transgen, Beijing, China) to generate sticky ends. Subsequently, gel extraction was performed using the Easy Pure Quick Gel Extraction Kit (EG101, Transgen, Beijing, China). The resulting fragments were then ligated to the pGL3-Basic plasmid using T4 DNA Ligase (FL101, Transgen, Beijing, China). Finally, transformation into bacteriophage was carried out using Trans1-T1 Phage Resistant Chemically Competent Cells (CD501, Transgen, Beijing, China), followed by plasmid extractions using Endofree MINI Plasmid kit II (DP118, TINGEN, Beijing, China). The resulting plasmids were named pGL −1855/+55 (P1), pGL −1629/+55 (P2), pGL −1329/+55 (P3), pGL −1029/+55 (P4), pGL −729/+55 (P5), pGL −429/+55 (P6), and pGL −129/+55 (P7).

### 2.5. Cell Culture, Transfection, and Luciferase Activity Analysis

The medium consisted of 10% fetal bovine serum (A6904, Invitrogen, Carlsbad, CA, USA) and 4% dual antibiotics (G4003-100ML, Servicebio, Wuhan, China), supplemented with 86% DMEM high glucose medium (C11995500BT, GIBCO, Grand Island, NY, USA). Mouse C2C12 cells (TCM-C720, Starfish Bio, Suzhou, China) were cultured in the above configured medium. Cells were seeded into 24-well plates at a density of 1 × 10^5^ cells per well prior to transfection and then cultured at 37 °C in a CO_2_ incubator with a concentration of 5% until reaching a cell density of 75–80%. For detection purposes, following co-transfection according to the manufacturer protocol from Invigentech (Invitrogen, Carlsbad, CA, USA), using the TransDetect Dual Luciferase Reporter Gene Assay Kit (FR201, Transgen, Beijing, China), cells were transfected with the target plasmid along with pGL3-TK at an ng ratio of 800:20 for a duration of 48 h. Subsequently, collected cells were passed through an enzyme labeler (VL0000D0, Thermo Scientific, Waltham, MA, USA). Firefly luciferase and Renilla luciferase activities were measured, and the promoter activity was determined as firefly luciferase activity/Renilla luciferase activity in three-replicate wells per transfection.

In accordance with this, bovine adult myoblasts were obtained from cells isolated and identified as having better activity by the previous group [23]. Their medium consisted of 13% FBS, 4% antibiotics, and 83% DMEM/F12 medium (C11330500BT, Hyclone, New York, NY, USA). Prior to transfection, cells were seeded into 12-well plates at a density of 1 × 10^6^ cells per well and then cultured in a CO_2_ incubator at 37 °C at a concentration of 5% until cell densities reached 75–80% for later RNA interference experiments.

### 2.6. Site-Directed Mutagenesis

The PCR amplification kit was utilized for the amplification, employing custom-designed glucocorticoid receptor (GR) and specificity protein 1 (SP1) sentinel mutation primers based on the Fast MultiSite Mutagenesis System (FM201, Transgen, Beijing, China) (Table S1). The reaction system comprised 25 μL of 2× TransStart FastPfu Fly PCR SuperMix, 1 μL each of the upstream and downstream primers, 9 μL of Plasmid, and finally 14 μL of Nuclease-free Water. The amplified target sequences were subsequently purified and assembled. The reaction was carried out at 50 °C for 15 min followed by rapid cooling on ice for a few secs. Subsequently, the receptor cells underwent transformation, and single clones were selected for sequencing.

### 2.7. RNA Interference Assay

The interfering sequences targeting transcription factor GR and Sp1 (siRNA-GR, siRNA-SP1) were custom-synthesized by Suzhou Hongxun Biotechnology Co. (Suzhou, China). The specific details of these interfering sequences are provided (Table S1).

### 2.8. Electrophoretic Mobility Shift Assay (EMSA)

To obtain nuclear protein extracts from mouse C2C12 cells, logarithmic growth phase cells were cultured into T25 culture flasks. A Nuclear Protein Extraction Kit (Active, Motif Corp, Carlsbad, CA, USA) was used for treating the C2C12 cells. Biotin-labeled DNA probes for the 5′UTR proximal core transcriptional regulatory region were designed and synthesized (Table S1). In brief, a binding reaction mixture consisting of 2 µL of 5× binding buffer, 10 µg of nuclear extract, and 1 µL of poly (dI-dC) was prepared in a volume of 20 µL and incubated on ice for 15 min. Subsequently, biotin-labeled DNA (200 fmol) was added to the reaction mixture and incubated at room temperature for an additional 20 min. For competition assays, unlabeled or mutant probes were added to the reaction mixture 15 min prior to adding the labeled probe. For the super-shift assay, antibodies (10 µg), including anti-GR (TA7647A, Abmart, Shanghai, China) and anti-SP1 (PS02143, Abmart, Shanghai, China), were included in the reaction mixture after pre-incubation at a low temperature for 30 min. Following this step, DNA–protein complexes were separated by non-denaturing polyacrylamide gel electrophoresis using polyacrylamide and a borate–EDTA buffer for a one-hour duration.

### 2.9. Statistical Analysis

The data are presented as mean ± SD from three independent experiments, and statistical analysis was performed using IBM SPAA 26.0 Multiple-group comparisons were conducted using one-way ANOVA, while two-group comparisons were analyzed using a two-tailed Student’s *t*-test.

## 3. Results

### 3.1. Sequence Structure, Homology Analysis, and Phylogenetic Tree Construction of the Bovine CDH11 Gene

**Figure 1:**
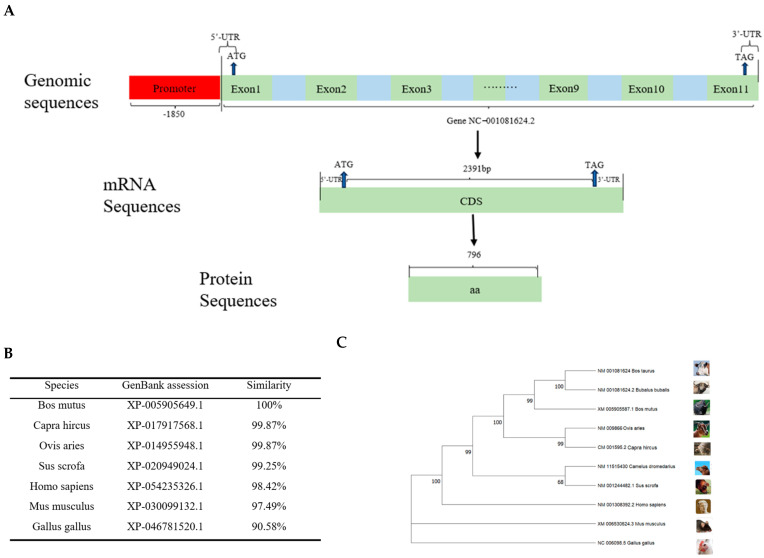
Structural homology and phylogenetic tree of the *CDH11* gene. (**A**) This section mainly includes the *CDH11* gene promoter, 5′UTR, 3′UTR, exons, introns, coding length of CDS region, and number of coding amino acids. (**B**) A comparison of the degree of amino acid clustering in the *CDH11* protein among seven different species. (**C**) An analysis of the protein–amino acid phylogenetic tree for the *CDH11* gene among 10 different species.

We characterized the bovine *CDH11* gene structure and discovered that it is located on chromosome 18 and contains 11 exons and 10 introns. The NC_001081624.2 mRNA transcript of this gene is 2391 bp, which can encode 796 amino acids (Figure 1A).

The *CDH11* gene sequences of seven different species were selected and compared with those of *Bos taurus* (XP_005218806.1). The results revealed that the similarity was 100% for *Bos mutus* (XP_005905649.1), while the similarity with *Capra hircus* (XP_ 017917568.1) and *Ovis aries* (XP_014955948.1) were both with 99.87%. Additionally, the similarity with *Sus scrofa* (XP_020949024.1), *Homo sapiens* (XP_054235326.1), *Mus musculus* (XP_ 030099132.1), and *Gallus gallus* (XP_046781520.1) were found to be 99.25%, 98.42%, 97.49%, and 90.58%, respectively (Figure 1B). It is evident from these findings that the *CDH11* gene exhibits a higher degree of conservation in ruminants compared to non-ruminants.

To comprehensively understand the evolution of the *CDH11* gene, we selected *Bos taurus* (NM_001081624), *Bos mutus* (XM_005905587.1), *Bubalus bubalis* (NM_001081624.2), *Capra hircus* (NM_009866), *Ovis aries* (CM_001595.2), *Camelus dromedarius* (NM_011515430), *Homo sapiens* (NM_001308392.2), *Mus musculus* (XM_006530624.3), *Sus scrofa* (NM_001244482.1), and *Gallus gallus* (NC_006098.5) using the EMGA11 software in conjunction with the amino acid sequences published by UCSC, with their *CDH11* amino acid sequences used for phylogenetic tree construction. The results showed that *Bos taurus* formed a cluster with ruminants, including *Bubalus bubalis*, *Bos mutus*, *Capra hircus*, *Ovis aries*, and *Camelus dromedarius*, followed by a cluster comprising non-ruminants such as *Sus scrofa*, *Homo sapiens*, and *Mus musculus* in another cluster, while *Gallus gallus* was the most distantly related group (Figure 1C).

### 3.2. Expression Assay of Bovine CDH11 Gene in Various Tissues

**Figure 2:**
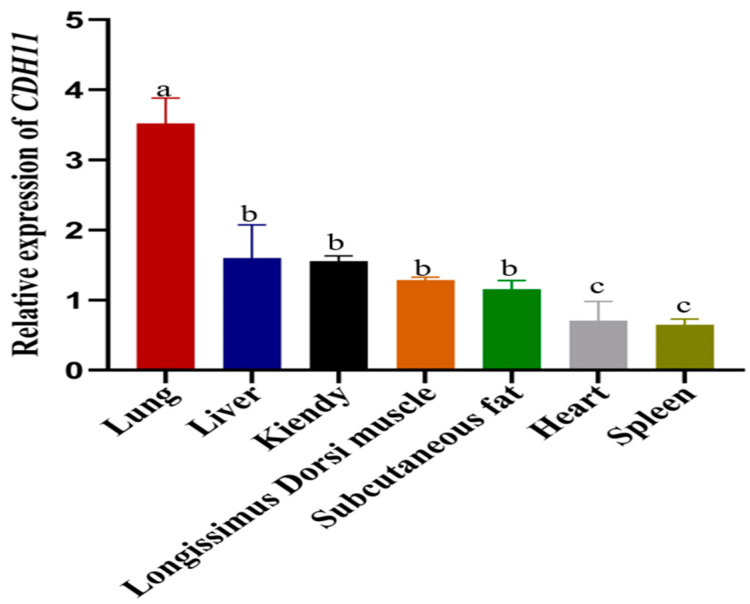
The expression of the *CDH11* gene in various bovine tissues was analyzed using qRT-PCR with the heart as a reference. The results are presented as the mean ± standard deviation (SD) of three experimental groups. Different letters indicate significant differences between groups, while the same letter indicates no significance. Error bars represent SD.

To investigate the mRNA expression pattern of the *CDH11* in various tissues, total RNA was extracted from the heart, liver, spleen, lung, kidney, *longissimus dorsi*, and subcutaneous fat. Subsequently, the isolated RNA was reverse-transcribed into cDNA. Quantitative real-time polymerase chain reaction (qRT-PCR) analysis revealed that the *CDH11* gene exhibited differential expression across different tissues with the lung display the highest expression. Moreover, moderate to high levels of *CDH11* gene expression were observed in the liver, kidney, *longissimus dorsi*, and subcutaneous fat, which were significantly compared to spleen and heart tissues. Notably, the spleen exhibited the lowest level of the *CDH11* gene expression (Figure 2).

### 3.3. Identification of the Core Transcriptional Regulatory Region of the Bovine CDH11 Gene

**Figure 3:**
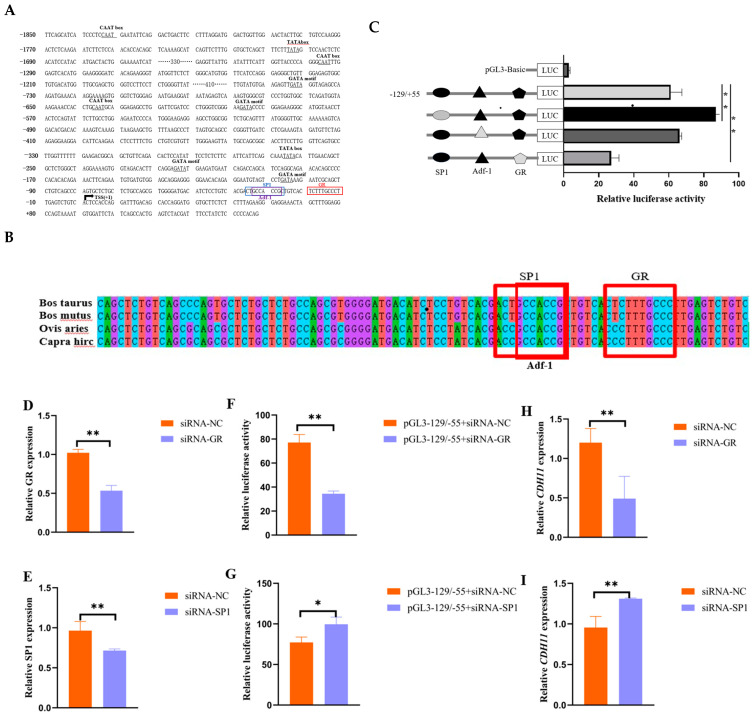
Effect of transcription factors SP1 and GR on the transcriptional activity and expression of the *CDH11* gene. (**A**) The promoter region of the *CDH11* gene (−1855/+55) sequence is represented, with the arrow TSS (+1) indicating the transcriptional start site. The GR binding site to the SP1 transcription factor is shown in the blue box, and predicted transcriptional regulatory elements are marked with black markers. (**B**) The −129/+55 vector sequence containing a mutant GR and SP1 binding site was constructed, and luciferase activity was measured by transfecting mouse C2C12 cells for 48 h using the −129/+55 vector plasmid as a negative control. The black and gray filled graphs represent wild type and mutant. (**C**) Conservation analysis of GR and SP1 in multiple species. (**D**,**G**) Detection of siRNA-GR and siRNA-SP1 interference efficiency in bovine adult myocytes using siRNA-NC as a negative control. (**E**,**H**) Luciferase activity was measured after co-transfection of bovine adult myoblasts with pGL3- 129/+ 55 + siRNA-NC for 48 h using pGL3 −129/+55 + siRNA-GR and pGL3 −129/+55 + siRNA-SP1 as negative controls. (**F**,**I**) Bovine adult myoblasts were transfected with siRNA-GR, siRNA-SP1 using siRNA-NC as a negative control, and *CDH11* gene expression was detected via qRT-PCR. * indicates *p* < 0.05, while ** indicates *p* < 0.01 compared to the control group.

To identify the core transcriptional regulatory region of the promoter region of bovine *CDH11* gene. We amplified seven segment-by-segment fragments from the 5′UTR proximal promoter region and successfully ligated NheI and XhoI restriction enzyme reporter fragments into a pGL3-Basic vector to construct a recombinant double-luciferase plasmid (Figure S1). Subsequently, the corresponding recombinant luciferase reporter plasmids were transfected into mouse C2C12 cells to assess luciferase activity. The results showed no significant change in luciferase activity compared to −1855/−429. Notably, luciferase activity was significantly higher in the −129/+55 region compared to the −429/+55 region, indicating elevated promoter activity compared to the pGL3-Basic vector plasmid (Figure 3A). In conclusion, our results suggest that the core transcriptional regulatory region of the bovine *CDH11* gene is located at −129/+55 bp relative to the TSS.

In addition, to investigate the potential impact of methylation on the expression of the promoter region of the *CDH11* gene, we used the online software tool Methprimer to predict the presence of CpG islands. Notably, the analysis showed the absence of CpG islands in the promoter region of the bovine *CDH11* gene, and therefore, we predicted that the expression of the *CDH11* gene may not be affected by methylation in the promoter region (Figure 3B).

### 3.4. The Validation of Transcription Factors in Core Regions of Transcriptional Regulatory

In an attempt to elucidate the role of potential trans-acting elements within the core promoter region of the bovine *CDH11* gene, this study aimed to predict its transcription factors using gene regulation and the Jaspar online platform. As a result, it was found that three transcription factor binding sites, SP1 tonic, ADF-1, and GR, which are associated with muscle growth and development, were present at the −36/27 bp, −34/−26, and −20/−11 bp sites in the core transcriptional regulatory region of the bovine *CDH11* gene (Figure 3A). In addition, conservation analyses showed that SP1, ADF-1, and GR exhibited significant conservation across multiple species (Figure 3B). To further investigate the functional significance of these three binding sites, vectors containing mutated core transcriptional regulatory regions of these two binding sites were constructed in this experiment. Interestingly, deletion of the SP1-binding site significantly increased luciferase activity, mutation of the ADF-1binding site resulted in no significant luciferase activity, and deletion of the GR-binding site resulted in a significant decrease in luciferase activity (Figure 3C).

Based on these findings, in the present study, siRNA-GR and siRNA-SP1 (Figure 3D,E), which have better interference efficiency, were co-transfected with the pGL3 −129/+55 plasmid, respectively, in bovine adult myoblasts, and their luciferase activities were determined. The results showed that the luciferase activity of siRNA-GR + pGL3 −129/+55 was significantly reduced, whereas that of siRNA-SP1 + pGL3 −129/+55 was significantly elevated, compared with that of the control (Figure 3F,G). It was also observed that knockdown of GR significantly inhibited the expression level of *CDH11*, whereas knockdown of SP1 significantly promoted the expression of *CDH11* (Figure 3H,I).

### 3.5. Electrophoretic Mobility Shift Assays (EMSAs) to Validate the Interaction of GR and SP1 with the Promoter

**Figure 4:**
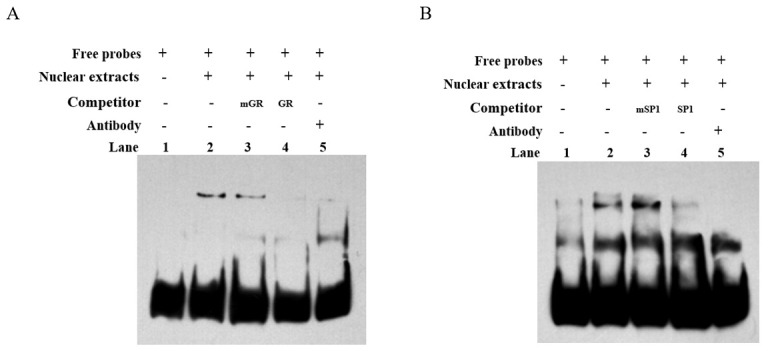
The EMSA confirmed the in vitro binding of transcription factors GR and SP1 to the core transcriptional regulatory region of the *CDH11* gene. (**A**) shows EMSA validation of transcription factor GR binding, while Figure (**B**) demonstrates the EMSA validation of transcription factor SP1 binding to the core transcriptional regulatory region of the *CDH11* gene. A biotin-labeled probe containing two transcription factors from the core transcriptional regulatory region of the *CDH11* gene was incubated with C2C12 cytosolic protein (lane 2), mutated probe (lane 3), and unmutated probe (lane 4); Sup remigration assays were performed using anti −GR and anti −SP1 antibodies (lane 5).

To confirm the binding of transcription factors GR and SP1 to the core transcriptional regulatory region of the bovine *CDH11* gene, we performed in vitro EMSA experiments. Biotin-labeled oligonucleotide probes GR and SP1 were synthesized and incubated with nucleoproteins from C2C12 cells. The biotin-labeled probes formed a DNA-protein complex band with nuclear proteins (Figure 4A,B lane 2). Addition of the noncompetitive probe had minimal impact on the DNA protein complex bands (Figure 4A,B lane 3), while the addition of a competitive probe resulted in attenuation and disappearance of the DNA protein complex bands (Figure 4A,B lane 4). Furthermore, inclusion of GR antibody and SP1 antibody individually attenuated the DNA protein complex band *and* led to the formation of a super shifted band above it (Figure 4A,B lane 5). These findings indicate that both transcription factors GR and SP1 bind to the core transcriptional regulatory region of the *CDH11* gene.

## 4. Discussion

The rate of growth in skeletal muscle plays a crucial role in determining the carcass weight of livestock. Elucidating the transcriptional regulation of genes related to skeletal muscle can serve as a theoretical foundation for breeding meat-producing livestock and poultry. For example, the *CDH11* gene was identified by GWAS and RNA-seq as a potential regulator affecting the growth and development of beef cattle, and single nucleotide polymorphisms in the *CDH11* gene were identified as being associated with growth and development in Qinchuan cattle [10,24]. In this study, we observed that *CDH11* gene mRNA is highly expressed in the longissimus dorsi muscle compared to cardiac expression, suggesting its potential role in regulating bovine skeletal muscle production.

To further elucidate the epigenetic regulatory role of the cattle *CDH11* gene in muscle growth and development, this study conducted an analysis on the promoter sequence of the bovine *CDH11* gene obtained from the UCSC database. Our analysis revealed that the promoter region of bovine *CDH11* lacks a traditional TATA box and CpG island structure, which concurs with previous investigations demonstrating limited presence of TATA boxes in mammalian gene promoters and 50% occurrence of CpG islands either inter or intra-genic ally [25,26]. Therefore, it can be speculated that the methylation of the promoter region may not affect transcriptional regulation of the bovine *CDH11* gene. Furthermore, employing a segment-by-segment deletion approach, we identified −129/+55 bp as the core transcriptional regulatory region of the *CDH11* gene expression. In our study, we predicted two transcription-factor-binding sites (SP1 and GR) in the core transcriptional regulatory regions, −36/−27 bp and −20/−11 bp, that are highly conserved across multiple species and associated with muscle development. To investigate their role further, mutated vectors containing mutated core transcriptional regulatory regions were constructed, and the luciferase activity was measured. Deletion of the SP1-binding site significantly increased the luciferase activity, while deletion of the GR-binding site significantly decreased it. Hence, these results suggest that SP1 and GR play a crucial role in mediating the transcriptional active of bovine *CDH11* gene.

The GR is ligand-dependent transcription factor belonging to the nuclear receptor superfamily, which plays an active role in all stages of muscle strength and development. Previous studies have demonstrated its essentiality in maintaining and establishing a quiescent state during the growth and development phase of satellite cells. Knocking down GR in myosatellite cells has been shown to increase the amount of circulating cells in the proliferative phase, ultimately leading to muscular atrophy [27]. During myofibrilla genesis, GR synergistically binds with MyoD at the GC-rich DNA corresponding element region of the promoter of the target gene, resulting in programmed expression of muscle-fiber-related genes [28,29,30]. Although MyoD has been identified as a specific transcription factor for skeletal muscle proliferation and differentiation, it may also act through alternative pathways or transcription factors on muscle precursors [28,31]. In this study, interference with the GR led to a significant decrease in luciferase activity within the core transcriptional regulatory region of the *CDH11* gene as well as a reduction in *CDH11* gene expression. Additionally, results from the EMSA demonstrated that the GR transcription factor could bind to the promoter region sequence of the *CDH11* gene. These findings suggest that GR transcription factors positively regulate the expression of the cattle *CDH11* gene in bovine muscle growth and development.

Transcription factor SP1, a member of the Sp/Kruppel superfamily, is an eukaryotic transcription factor that has been identified to directly regulate mammalian muscle growth and development [32,33]. Previous studies have indicated that the SP1 recruits MyoD, MyoG, and MEF-2C elements to specifically bind to GC-box and GT-box progenitors in gene promoters for the regulation of myosatellite cell proliferation and differentiation [34,35]. In this study, mutation of the SP1 binding site significantly enhanced the transcriptional activity of the core transcriptional regulatory region of the *CDH11* gene. Overexpression of the *CDH11* gene has been shown to inhibit MyoD and MyoG expression [13]. Therefore, we hypothesize that the SP1 specifically binds to the promoter region of *CDH11* gene and plays a role in regulating bovine myoblastproliferation and differentiation by recruiting MyoD, MyoG, MEF-2C, or itself. Ultimately, this positively impacts bovine myogenesis [36,37]. Interference with the SP1 transcription factor led to increased luciferase activity in the core regulatory region of *CDH11* gene, resulting in reduced levels of *CDH11* gene expression. EMSA results confirmed that the SP1 can bind to the core regulatory region of the *CDH11* gene. In brief, our study demonstrates that the transcription factor SP1 plays a crucial role in regulating *CDH11* gene expression in bovine skeletal muscle. However, further investigation is required to understand the epigenetic modifications with its promoter as well as its specific transcriptional regulatory mechanisms related to the *CDH11* gene function. These findings provide insights into understanding how the bovine *CDH11* gene is regulated at a transcriptional level and its biological function.

## 5. Conclusions

In conclusion, this study establishes that the *CDH11* gene is highly expressed in bovine longissimus dorsi muscles and identifies its core promoter region within −129/+55 bp. Our results suggest that *CDH11* is positively regulated by GR and negatively regulated by SP1 in the regulation of muscle growth and development. These findings enhance our understanding of the transcriptional regulation mechanisms governing the *CDH11* gene and suggest potential molecular breeding strategies for improving beef cattle yield and quality.

## Data Availability

Sequence data that support the findings of this study have been deposited in the NCBI with the primary accession code PQ364140 1.

## Supplementary Materials

The following supporting information can be downloaded at https://www.mdpi.com/article/10.3390/ani15091217/s1; Figure S1: Agarose gel electrophoresis of the original deletion promoter fragment. Figure S2: Original EMSA figures; Table S1: The primer sequences for RT-PCR, qRT-PCR, Promoter cloning, site-mut, EMSA and interference.
