# Supplementary material for: Cloning and Transcriptional Activity Analysis of the Bovine CDH11 Gene Promoter: Transcription Factors Sp1 and GR Regulate Bovine CDH11 Expression
Source: Animals (Basel). 2025 Apr 25;15(9):1217. doi: 10.3390/ani15091217 (PMC12071067; doi:10.3390/ani15091217)
Supplement: Supplementary file 1 [file animals-15-01217-s001.zip › Table S1.pdf]

Supplementary Information

Supplementary Table S1.The primer sequences for RT-PCR, qRT-PCR, Promoter cloning,site-mut,EMSA and interference

Item	Primer name	Primer sequences（5′→3′）
RT-PCR	CDH11	F: TTTACAAGCCGCCCTGGTG
		R: CCGTACAAGTCCGCTAGTTTC
	SP1	F: AACGGCAACAATGGCAGTGAG
		R: GCTGGTTCTGAAGGTTGGAAGTG
	GR	F: AGGAGGATGACGAAGAGGATGAAG
		R: AGCCACGACACCCGAACTC
	GAPDH	F: AAGTTCAACGGCACAGTCA
		R: GTCATAAGTCCCTCCACGAT
Promoter cloning	pGL-1855/+55(P1)	F: CCAAGGGACTCTCAAGAATC
		R: TTTACTGGCCTCCAAAGC
	pGL-1629/+55(P2)	F: GGCTGCAGTCACCATCTGCA
		R: TTTACTGGCCTCCAAAGC
	pGL-1329/+55(P3)	F: GGGCATGTGGTTCATCCAGG
		R: TTTACTGGCCTCCAAAGC
	pGL-1029/+55(P4)	F: GGGCTACAGTCCATGGGGTT
		R: TTTACTGGCCTCCAAAGC
	pGL-729/+55(P5)	F: AAGAAACCACCTGCAATGCAGG
		R: TTTACTGGCCTCCAAAGC
	pGL-429/+55(P6)	F: ACCTTCCTTGGTTCAGTGCCT
		R: TTTACTGGCCTCCAAAGC
	pGL-129/+55(P7)	F: ATCTCCTGTCACGACTGCCAC
		R: TTTACTGGCCTCCAAAGC
Site-mut and EMSA	SP1	F: CCAGTGCTCTGCTCTGCCAGCGTGGGGATGACATCTCCTGTCACGACTGCCACCGCTGT
		R: ACAGCGGTGGCAGTCGTGACAGGAGATGTCATCCCCACGCTGGCAGAGCAGAGCACTGG
	mSP1	F: CCAGTGCTCTGCTCTGCCAGCGTGGGGATGACATCTCCTGTCACGAGAGGGTGGAGTGT
		R: ACACTCCACCCTCTCGTGACAGGAGATGTCATCCCCACGCTGGCAGAGCAGAGCACTGG
	GR	F: TGTCACTCTTTGCCCTTGAGTCTGTCACTCCACCAGGATTTGACAGCACCAGGATGGTG
		R: CACCATCCTGGTGCTGTCAAATCCTGGTGGAGTGACAGACTCAAGGGCAAAGAGTGACA
	mGR	F: TGTCACAGAACCGGTATGAGTCTGTCACTCCACCAGGATTTGACAGCACCAGGATGGTG
		R: CACCATCCTGGTGCTGTCAAATCCTGGTGGAGTGACAGACTCATACCGGTTCTGTGACA
interference	siRNA-GR	F: GGUGCAGACAACCAGAUUACAdtdt
		R: UGUAAUCUGGUUGUCUGCACCdtdt
	siRNA-SP1	F: GGAUGGUUCAGGUCAGAUACAdtdt
		R: UGUAUCUGACCUGAACCAUCCdtdt
