# Supplementary material for: Cloning and Transcriptional Activity Analysis of the Bovine CDH11 Gene Promoter: Transcription Factors Sp1 and GR Regulate Bovine CDH11 Expression
Source: Animals (Basel). 2025 Apr 25;15(9):1217. doi: 10.3390/ani15091217 (PMC12071067; doi:10.3390/ani15091217)
Supplement: Supplementary file 1 [file animals-15-01217-s001.zip › Figure S1.pdf]

**Supplementary Materials:**

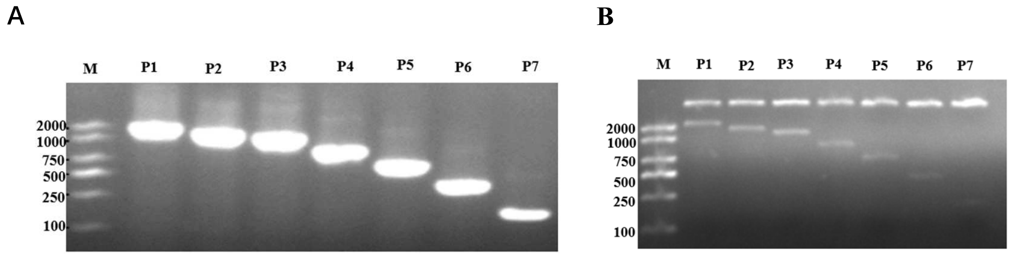


Figure S1. Agarose gel electrophoresis of the original deletion promoter fragment. (A) Different lengths of fragments of *CDH11* promoter cloned by RT-PCR. (B) Detection of vectors construction of *CDH11* promoter in different lengths fragments. M: marker, P1: PGL-1855/+55, P2: PGL-1629/+55, P3: PGL-1329/+55, P4: PGL-1029/+55, P5: PGL-729/+55, P6: PGL-429/+55 and P7: PGL-129/+55.
