# Supplementary figures and images for: Cloning and Transcriptional Activity Analysis of the Bovine CDH11 Gene Promoter: Transcription Factors Sp1 and GR Regulate Bovine CDH11 Expression
Source: Animals (Basel). 2025 Apr 25;15(9):1217. doi: 10.3390/ani15091217 (PMC12071067; doi:10.3390/ani15091217)

**Supplementary Materials:**

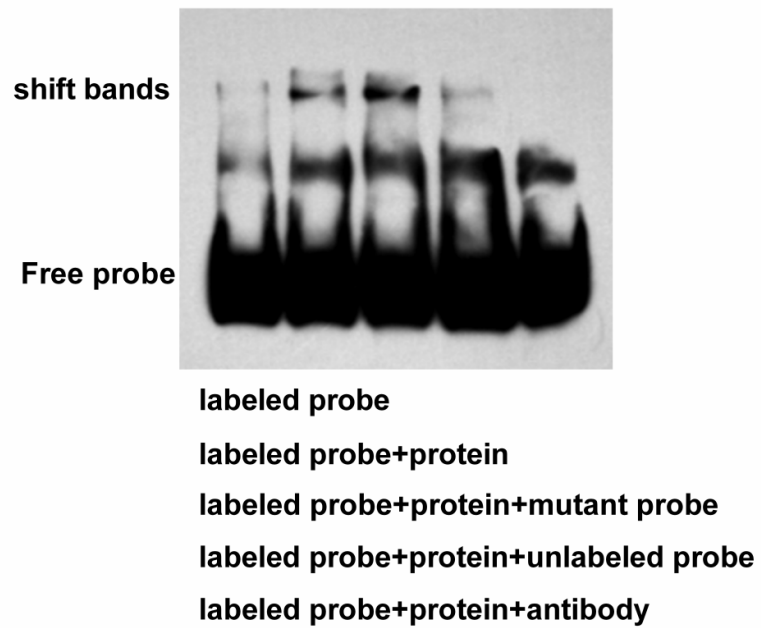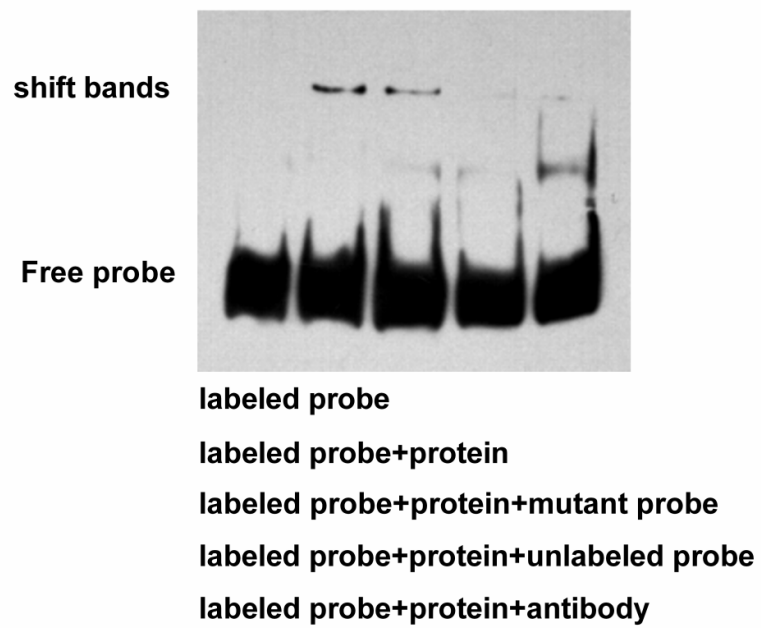

**Figure S2. Original EMSA figures**

Supplement: Supplementary file 1 [file animals-15-01217-s001.zip › Figure S2.Original EMSA figures.pdf]
